# Dioptric blur is not fully reflected by VEP-based visual acuity estimates

**DOI:** 10.1007/s10633-025-10044-z

**Published:** 2025-07-31

**Authors:** Dillys A. D. Amega, Julia Haldina, Ingrid Toews, Heiko Philippin, Michael B. Hoffmann, Enyam K. A. Morny, Sven P. Heinrich

**Affiliations:** 1 Department of Vision Science, School of Optometry and Vision Science, University of Cape Coast, Cape Coast, Ghana; 2 Eye Center, Medical Center, University of Freiburg, Killianstr. 5, 79106 Freiburg, Germany; 3 Medical Center, Institute for Evidence in Medicine, University of Freiburg, Freiburg, Germany; 4 Faculty of Medicine, University of Freiburg, Freiburg, Germany; 5 Faculty of Infectious and Tropical Diseases, International Centre for Eye Health, London School of Hygiene and Tropical Medicine, London, UK; 6 Department of Ophthalmology, Otto-von-Guericke University, Magdeburg, Germany; 7 Center for Behavioral Brain Sciences, Magdeburg, Germany

**Keywords:** Visual acuity, Visual evoked potentials, Defocus, Dioptric blur, Gaussian blur, Spurious resolution, Grating acuity

## Abstract

**Purpose:**

Objective estimation of visual acuity (VA) based on visual evoked potentials (VEPs) has become an established technique for cases where psychophysical VA might be unreliable. Refractive errors and improper accommodation could undesirably affect the outcome of VA measurements. Consequently, it is of interest whether a VA reduction due to dioptric blur is reflected by VEP-based estimation of VA.

**Methods:**

We degraded vision in 19 participants to nearly 1.0 logMAR by using either plus lenses or a filter that creates Gaussian blur. For both types of degradation, we compared the outcomes of objective VEP-based VA testing to standard psychophysical VA. For comparison, we also obtained psychophysical grating VA.

**Results:**

With Gaussian blur, both values, VEP-based VA and psychophysical Landolt-C VA, were nearly identical. With dioptric blur, VEP-based VA was better than psychophysical Landolt-C VA in all participants by an average of 0.37 logMAR with some interindividual variability. Psychophysical grating VA was only relatively mildly affected by blur with no sizable differential effect of blur type.

**Conclusion:**

VEP-based estimation of VA does not reveal the full amount of VA reduction in the case of dioptric blur. On the one hand, this decreases VEP-based methods’ susceptibility to incorrect refraction and mis-accommodation, which are not normally the targeted causes of VA reduction. On the other hand, it reduces the accuracy in quantifying refraction-related impairments of vision with VEPs.

## Introduction

Standard “subjective” visual acuity (VA) testing employs psychophysical techniques with optotype charts or with partially automated methods, such as the Freiburg Visual Acuity and Contrast Test (FrACT) [1]. An excellent VA outcome is often taken as an indication of the integrity of the ocular media, clear image formation on the retina, healthy afferent visual pathway and functional stimulus processing at the visual cortex [2]. Clinically, a person’s VA is measured by asking the patient to discriminate and report optotypes of known angular dimensions [3]. This makes the method unreliable if the patient does not cooperate. Procedures based on visual evoked potentials (VEPs) have been established as an alternative method to assess VA objectively, for instance in non-verbal infants, adults with low intellectual abilities, or malingering [4, 5].

Different types of disease are associated with different pathophysiological changes that might affect psychophysical optotype VA and VEP-based measures of VA differentially. An example for this is amblyopia, where grating VA and VEP have been shown to overestimate VA [6, 7]. A dissociation between psychophysical optotype VA has also been reported for the case where vision had been impaired by artificially inducing fragmentation and distortion through the use of patterned polymethyl methacrylate sheets [8]. The present study aims at addressing this issue for two types of visual degradation that are seemingly more similar, namely Gaussian blur and dioptric blur [9]. The term “Gaussian” refers to the shape of the point-spread function or blur kernel that is associated with this type of blur, while the term “dioptric” refers to the refractive error (naturally existing or artificially created) that causes the blur.

Generally, blur reduces retinal contrast, in particular at higher spatial frequencies, which results in a reduction in VA. If the contrast reduction is sufficiently strong, the VEP will also be reduced. With non-gaussian blur, including dioptric blur, the relationship between spatial frequency and contrast reduction can be relatively complex, with extinguished and persisting (albeit reduced and potentially reversed) contrast alternating when spatial frequency is more and more increased. This phenomenon of contrast reappearing at higher frequencies is known as spurious resolution [9, 10] and might manifest as a perceived distortion of stimulus structure if multiple spatial frequencies are present.

The differences between Gaussian and dioptric blur have manifested in previous studies. Although dioptric blur created by the introduction of plus lenses in front of a patient’s eyes has been shown to achieve reduction in VA in many studies, only a relatively reduced effect on grating VA has been reported [11–13]. This has been proposed to result from spurious resolution, which would allow the orientation of a grating stimulus to be judged at relatively high levels of blur. However, the observer cannot make sense of the distorted image of an optotype that results from spurious resolution [9]. VEP-based techniques of VA testing are potentially susceptible to the effects of spurious resolution because the affected defocused stimulus pattern will still be able to evoke a VEP response, while an optotype seen with the same amount of dioptric blur might become unrecognizable. It would therefore be expected that VEP-based VA values should be better than psychophysical optotype-VA values. However, there is little specific empirical evidence for this yet. Using similar stimuli for both VEP-based VA and psychophysical grating VA, Tyler et al. [14] reported in a “good observer” under conditions of strong dioptric blur that VEP-based estimates were worse than psychophysical grating VA. Petersen [15] presented a technique to determine the refractive error through a VEP-based technique. Although that study implies a systematic impact of different amounts of dioptric blur on VEP amplitude at a fixed checker size of 15 arcmin, VEP-based estimates of VA were not obtained. There are, of course, several further studies that assessed the effect of dioptric blur on the VEP response to suprathreshold check sizes (e.g., [16–18]), which is relevant for certain applications other than VA estimation. In the present study, we set out to specifically assess how dioptric blur is reflected by VEP-based VA estimates, with Gaussian blur used for comparison.

## Methods

Twenty healthy participants (11 female) in the age range of 19–39 years (average 27 years) with best corrected VA of 0.0 logMAR or better and no known ophthalmological or neurological disorders were recruited for this study. All provided written informed consent. The study belonged to a larger project that was approved by the University of Freiburg's institutional review board. One participant was excluded from analysis because of a protocol violation.

The general concept of the study was to compare gaussian and dioptric blur in such a combination that both sources of blur produced (nearly) the same amount of psychophysical optotype VA, while the effects on psychophysical grating VA and on VEP-based VA were assessed. Gaussian blur was created using a Luminit 0.5° “light shaping diffusor” filter (Luminit, Torrance, CA, USA) [19], and dioptric blur was induced with plus lenses that were selected for each participant individually such that optotype VA was nearly identical for both types of blur.

### Psychophysical testing

All psychophysical VA testing was performed using the Freiburg Acuity and Contrast Test (FrACT) [1] on a 5-inch smallHD 501 monitor (SmallHD Inc., Cary, NC) at a distance of 57 cm. Optotype VA was determined with Landolt Cs [20] using 18 trials per test run, and with square wave gratings extending over 11° × 6.2° using 24 trials per run. Participants responded by pressing corresponding buttons on a response device. FrACT was used with the “DIN/ISO correction” (adjusting the outcome value to match values as obtained with a VA chart) for the Landolt C task. The grating VA outcomes were adjusted accordingly after converting the resulting threshold value (cycles per degree, cpd) into logMAR values following the common convention that 30 cpd correspond to a logMAR value of zero (in other words, when the stripe width of the grating and the gap width of the Landolt C are identical, the same logMAR value is obtained).

### VEP-based acuity estimation

In agreement with the respective ISCEV extended protocol [21], we used the technique described by Bach et al. [22]. In short, steady-state responses were obtained from three electrodes placed at the occipital pole in a Laplacian montage with a frontal reference electrode. Stimuli were checkerboard patterns with six different check sizes, which were presented on a CRT monitor (FIMI-Philips, Saronno, Italy) in a pattern-pulse style at 7.5 Hz and the respective noise-corrected response amplitudes were determined. In the resulting tuning curve (response amplitude vs. check size), the descending slope towards smaller check sizes was identified through a heuristic algorithm, and a straight line was fitted and extrapolated to find the check size corresponding to zero amplitude. The resulting value was converted into a VA estimate using an empirical conversion factor.

### Procedure

#### Preparation

Refractive status was determined with a Nidek AR-1 s autorefractor (Nidek Co. Ltd., Gamagori, Japan) followed by subjective refraction. Best corrected VA with a near addition of + 1.75 D to account for the testing distance of 57 cm (identical for psychophysics and VEP recordings) was used as a basis for all subsequent manipulations of the participant’s vision. Following binocular practice runs with both Landolt Cs and gratings, Landolt-C VA was determined for each eye and the eye with better VA was chosen as the study eye, with which all the subsequent steps were performed. The other eye was occluded.

#### Psychophysical testing

Whenever the Luminit filter (Gaussian blur) or the corresponding plus lenses (dioptric blur) were placed in front of the study eye, participants were allowed two minutes of adaptation during which they could look around freely. First, Gaussian blur was used. Landolt-C VA and grating VA were both obtained twice, and the respective logMAR averages (VA_GaussL_ and VA_GaussGr_) were computed.

With the Luminit filter removed, positive lenses (dioptric blur) were then given to obtain a similar VA as with the Luminit filter (Gaussian blur). The dioptric blur VA was considered similar to Gaussian blur VA if the difference in standard VA was not more than 0.2 logMAR. With one exception of + 4.00 D, this was achieved with lenses with an optical power *P* of + 3.00 or + 3.50 D, matching typical values for the VA range [23]. Both Landolt-C VA and grating VA were subsequently obtained twice, and again the respective logMAR averages were recorded (VA_DiopL_ and VA_DiopGr_). In case of a Landolt-C VA difference of more than 0.2 logMAR between measurements with Luminit filter vs plus lenses, the power of the plus lenses was re-adjusted, and the VA measurements were repeated.

#### VEP-based acuity estimation

Participants were then set up for the VEP measurement. With each type of blur, two measurements were performed using an ABBA scheme with the assignment of A and B to Gaussian and dioptric blur alternated between participants, i.e., if one participant started with the Luminit filter, the next one would start with the plus lenses. Again, the corresponding logMAR VA values were averaged, yielding VA_GaussVEP_ and VA_DiopVEP_.

Finally, a standard transient VEP [24] without degradation was measured to confirm normal visual pathway function.

## Analysis

All Data analysis was performed using IGOR Pro 8 (Wavemetrics Inc., Lake Oswego, USA).

### Per-participant analysis

For both types of blur, the respective psychophysical Landolt-C VA was taken as reference value. The deviation of psychophysical grating VA and VEP-based VA relative to the reference was computed by subtracting the reference value (Landolt-C VA) from the other value, yielding ∆VA_GaussGr_ and ∆VA_GaussVEP_ as well as ∆VA_DiopGr_ and ∆VA_DiopVEP_ for each participant. Values that are more negative indicate that the respective values for grating VA and VEP-based VA are better than those for Landolt-C VA.

### Group-level analysis

The null hypotheses were ∆VA_GaussGr_ ≤ ∆VA_DiopGr_ and ∆VA_GaussVEP_ ≤ ∆VA_DiopVEP_ (with VA given as logMAR). They were directional because of the assumed presence of spurious resolution with dioptric blur but not with Gaussian blur, which let us predict better VEP-based acuity values with dioptric blur. The hypotheses were tested using paired one-sided permutation tests (n = 10,000). 95% confidence intervals (CI_95_) were obtained through bootstrapping.

## Results

**Fig. 1:**
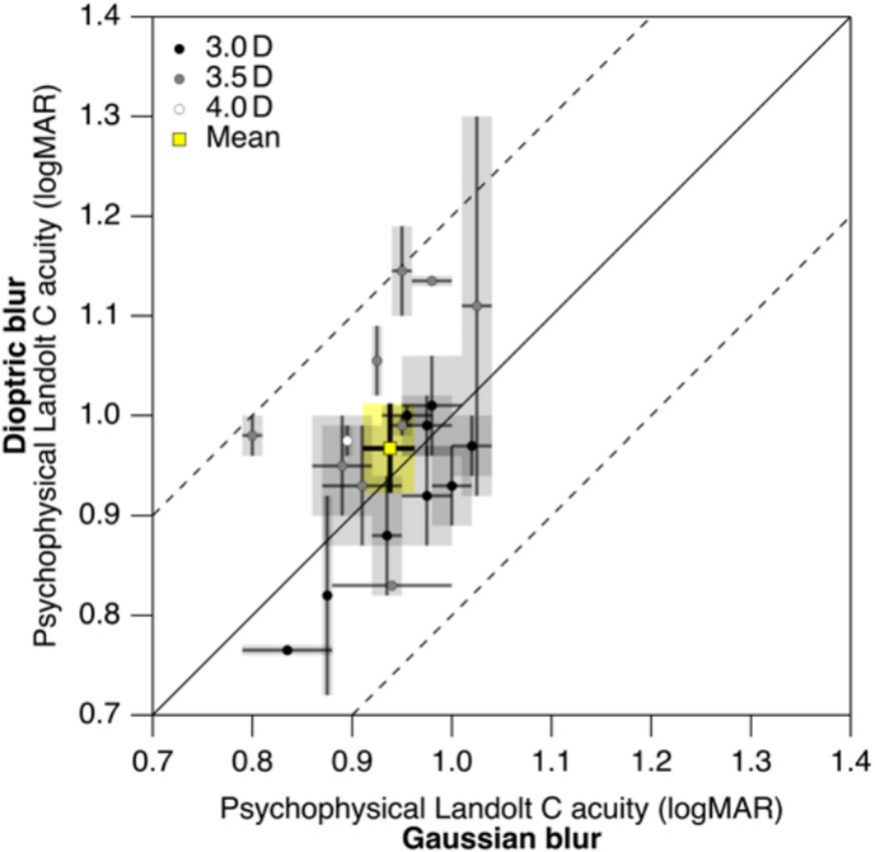
Comparison of psychophysical Landolt-C VA for both blur types. There is no sizable systematic difference. Two test runs were performed for each blur type. Circles represent the average, and the ends of the respective bars represent the individual outcomes of the two test runs. The difference in VA between dioptric and Gaussian blur did not exceed ± 0.2 logMAR (indicated by the dashed lines), as stipulated by the study protocol. Black, grey, and white filling of the markers represents the interindividually different lens power that was required to achieve the desired level of dioptric blur. The mean across participants is indicated by the square yellow marker, with the associated bars representing the 95% confidence intervals

As intended, Gaussian blur and dioptric blur resulted in similar average psychophysical Landolt-C VAs (0.94 logMAR, CI_95_ = [0.91, 0.96] and 0.97 logMAR, CI_95_ = [0.92, 1.01], respectively; Fig. 1). The mean difference was 0.029 logMAR (CI_95_ = [− 0.013, 0.074]). With higher lens power (*P* ≥ 3.5 D), VA tended to be slightly worse than VA obtained with the Luminit filter, and they tended to be slightly better with lower lens power (*P* = 3.0 D).

**Fig. 2:**
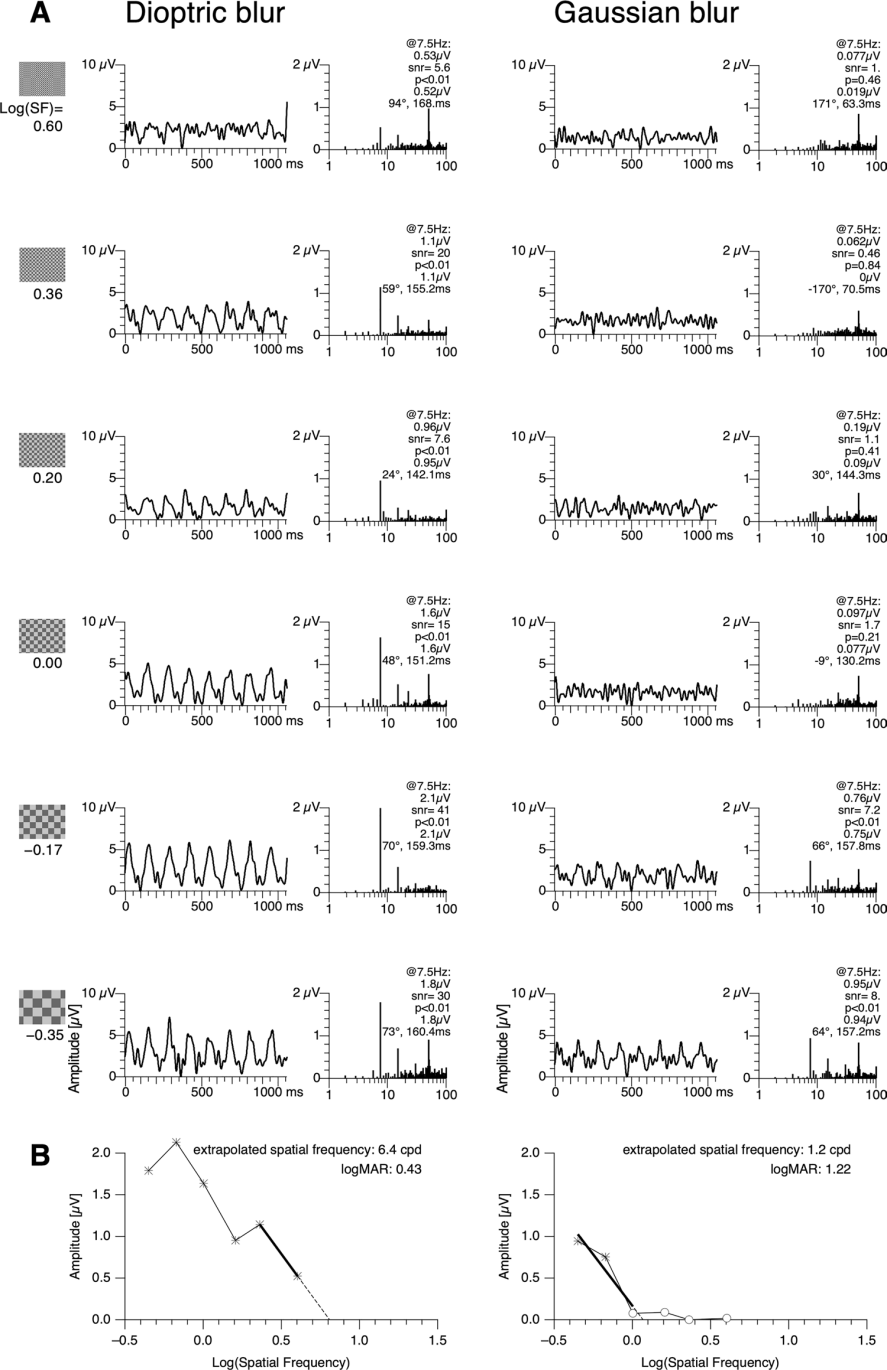
Comparison between dioptric blur and Gaussian blur for one exemplary participant. **A** Time series data and the corresponding Fourier spectra for 6 different check sizes from small (top) to large (bottom). For dioptric blur, responses are found also at small and medium check sizes. In contrast, with Gaussian blur only the two largest check sizes evoked significant responses. Time series data were digitally low-passed at 45 Hz for presentation. **B** Corresponding tuning curves, relating Fourier amplitude at the stimulation frequency to spatial frequency as determined by check size. The tuning curve for dioptric blur (left) extends to relatively high spatial frequencies (small checks), while the tuning curve for Gaussian blur is limited to low spatial frequencies (large checks)

Gaussian blur had the expected strong impact on the VEP and typically abolished responses to small and medium checks, as shown for one exemplary participant in Fig. 2A. In contrast, with dioptric blur the same participant produced responses to small and medium checks. This difference between conditions is reflected by the resulting tuning curves (Fig. 2B).

**Fig. 3:**
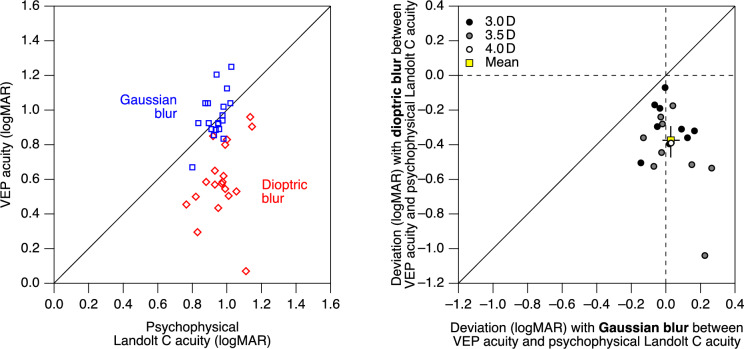
Left, individual VEP VAs plotted against the respective standard psychophysical Landolt C VAs. With Gaussian blur (blue squares), VEP VA matches psychophysical Landolt C VA quite well. In contrast, with dioptric blur (red diamonds), VEP VA is better (smaller logMAR values) than psychophysical Landolt C VA. Right, deviation of VEP VA of individual participants from psychophysical Landolt C VA, both obtained either with dioptric blur (vertical axis) or with Gaussian blur (horizontal axis). The yellow marker represents the mean values, with error bars indicating the 95% confidence interval. There is little deviation between VA measurements for Gaussian blur (the values are around zero), while there is a considerable systematic deviation of VEP VA towards smaller logMAR values for dioptric blur (the values are all less than zero). Black, grey, and white filling of the markers represent the interindividually different lens power required to achieve the desired level of dioptric blur

This outcome pattern was found for all participants. With Gaussian blur, VEP-based VA was generally very similar to psychophysical Landolt-C VA. With dioptric blur, on the other hand, VEP-derived VA values were systematically better than psychophysical Landolt-C VA (Fig. 3, left).

The experimental protocol allowed for small discrepancies in Landolt-C VA between Gaussian and dioptric blur. We accounted for this by subtracting from the VEP VAs the respective Landolt C VAs as obtained with the same type of blur, and then compared the resulting differences (Fig. 3, right). While both VA measurements closely agree in the presence of Gaussian blur (average logMAR deviation, 0.028; CI_95_ = [− 0.019, 0.080]; *p* = 0.60), there is a considerable discrepancy with dioptric blur in most participants (average logMAR deviation, − 0.37; CI_95_ = [− 0.47, − 0.29]; *p* < 0.0001). There is no statistically significant dependence of this effect on the lens power used to achieve the dioptric blur (spearman correlation, *ρ* = 0.38, *p* = 0.10; test for inequality of discrepancies, *P* = 3.0 D vs. *P* ≥ 3.5 D, *p* = 0.081).

**Fig. 4:**
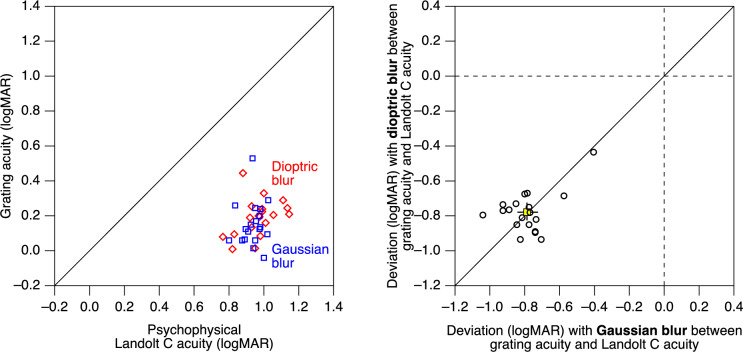
Left, individual psychophysical grating VAs plotted against the respective standard psychophysical Landolt C VAs. With both Gaussian blur (blue) and dioptric blur (red), grating VAs were better (smaller logMAR values) than Landolt C VAs by a similarly substantial amount. Right, deviation of grating VA of individual participants from Landolt C VA, both obtained either with dioptric blur (vertical axis) or with Gaussian blur (horizontal axis). The yellow marker represents the mean values, with error bars indicating the 95% confidence interval. While there is a considerable and fairly consistent deviation from Landolt C VA (i.e., values different from zero), there is not much difference between dioptric blur and Gaussian blur (data points cluster around the identity line)

Psychophysical grating VA was analyzed in an equivalent manner. With both types of blur, grating VAs were substantially better, by a similar amount, than Landolt C VAs (Fig. 4; average logMAR deviation between grating and Landolt-C VA with Gaussian blur, − 0.79; CI_95_ = [− 0.84, − 0.72], *p* < 0.0001; with dioptric blur, − 0.78; CI_95_ = [− 0.83, − 0.72], *p* < 0.0001).

## Discussion

The present study demonstrates a mismatch between psychophysical Landolt-C VA and checkerboard-based VEP VA in the presence of dioptric blur, with VEP-based values being better (smaller logMAR values). In contrast, no such discrepancy was found with Gaussian blur. A likely explanation is spurious resolution which results in stimulus structures remaining visible despite the pattern being substantially degraded by dioptric blur [9].

While the direction of this mismatch was consistent across participants, the amount was quite variable. Tentatively, we assume that this might be related to interindividual differences in VEP sensitivity to very low contrasts at which the spurious stimulus structures appear in the case of dioptric blur. Souza et al. [25] demonstrated such variability in contrast sensitivity for transient onset/offset VEPs. Although we did not address the case of improper accommodation directly, the present results imply an interindividually variable impact on the resulting VA estimate. This is particularly relevant in the context of intentional control of accommodation to induce defocus in malingering.

It seems plausible that the factors that cause better VA estimates with dioptric blur could also have a qualitative impact on the tuning curves that relate VEP amplitude to spatial frequency. In particular, spurious high spatial frequencies have a very low contrast and therefore the VEP would be expected to have small amplitudes in the respective spatial frequency range, even when accounting for the non-linear relationship between VEP amplitude and contrast [25]. Intriguingly, this is not what we found. Rather, amplitudes were relatively similar.

Somewhat unexpectedly, psychophysical grating VA did not show a differential effect of the type of blur. It was much less affected by either type of blur than was Landolt-C VA. The discrepancy between stimulus types is qualitatively consistent with previous findings with, e.g., Snellen letters and gratings, where particularly large disparities are found for low VA levels [12, 26, 27]. In the case of dioptric blur, several authors have related these disparities to spurious resolution that would prevent stimuli from becoming completely invisible at high levels of blur [10, 12, 28]. This explanation would not hold in the case of Gaussian blur, given the absence of spurious resolution [9].

In conclusion, a reduction of standard VA due to dioptric blur is not fully reflected by VEP-based VA. In some cases, this could be advantageous as it reduces the VEP method’s susceptibility to incorrect refraction and improper accommodation, which are not normally the targeted causes of VA reduction.

## Data Availability

Data is available upon request from the corresponding author.

## Declarations

### Human and animal participants

All procedures in studies involving human participants were in accordance with the ethical standards of the institutional and/or national research committee and with the 1964 Declaration of Helsinki and its later amendments or comparable ethical standards. This article does not contain any studies with animals other than *homo sapiens* performed by any of the authors.
